# Supplementary material for: KCTD Proteins Have Redundant Functions in Controlling Cellular Growth
Source: Int J Mol Sci. 2024 May 3;25(9):4993. doi: 10.3390/ijms25094993 (PMC11084553; doi:10.3390/ijms25094993)

Supplementary Figure Legends

Supplementary Figure S1. *GNB1 knockout confirmed by western blotting*. Immunodetection of the protein Gβ1 by western blot from cell lysates of the parental and the GNB1-KO cell lines (see *Material and Methods*) (top panel); the membrane was stripped of the anti-Gβ1 antibody and re-probed with an anti-β-tubulin used as a loading control (bottom panel); the arrow indicates the electrophoretic position of Gβ1. Data are representative of several experiments.

Supplementary Figure S2. *Genotyping analysis of the knockout cell lines*. A) Genomic DNA from the parental and GNB1 KO cell lines was extracted and a region of the GNB1 gene surrounding the sgRNA was amplified by PCR as described in *Materials and Methods* and the corresponding PCR products subjected to migration into an 1% agarose gel along with a molecular weight DNA ladder; numbers on the left side of the gel indicate the molecular size of the DNA fragments from the DNA ladder, the arrows on the right side are pointing to the PCR products and numbers indicate the relative size. B) Allele analysis of KTCD knockout lines by RFLP, as described in *Materials and Methods*, was performed for all the 3 target genes in parental as well as each of the 3 KO cell lines in order to report the editing progress during the sequential generation of the KO cell lines; at the top of the gel, amplified gene refers to the target KO gene and the restriction enzyme used to digest the DNA is in parentheses while the minus and plus signs indicate the absence or the presence of the corresponding enzyme treatment respectively and, located at the bottom of the gel are the cell lines that were genotyped: PL is parental, D2 is the KCTD5 KO, 2H6 is the double KCTD2/5 KO and 2B1 is the triple KCTD2/5/17 KO. The two main molecular weight markers are indicated at the left side of the gel (all other molecular weight markers are the same as in A). C) DNA sequence alignment between the WT and the edited alleles from each KO cell lines. DNA sequencing of PCR amplicons was done as described in *Materials and Methods* and

the sequencing results were aligned using Clustal Omega (Madeira, F., et al., (2022) NAR doi.org/10.1093/nar/gkac240) and then visualized using Jalview 2.11.3.2 (Waterhouse, A.M., et al (2009) Bioinformatics doi: 10.1093/bioinformatics/btp033). The description of each allele is indicated at the beginning of each sequence and the sgRNA sequence is indicated by a black bar; note that due to the large deletion that encompass the sgRNA sequence, no bar is shown for KCTD2/5 KO.

Supplementary Figure S 3. PCA depicting the principal component analysis results of gene expression data from two biological replicates of A) KCTD-KO lines compared to the parental line or B) the GNB1-KO line compared independently to the parental line, following DESeq2 analysis, illustrating sample relationships based on gene expression profiles of two independent biological replicates.

Supplementary Figure S4. *Understanding changes in gene expression in the KCTD knockout lines.* Gene ontology analysis was conducted to identify fold enrichments in pathways associated with differentially expressed genes across all KO cell lines compared to the parental line. The circular nodes in the figure represent distinct pathways, with the size of each circle indicating the number of identified genes within that pathway. False discovery rates (FDR) were graphically represented, providing insight into the statistical significance of pathway enrichments.

Supplemental Figure S1

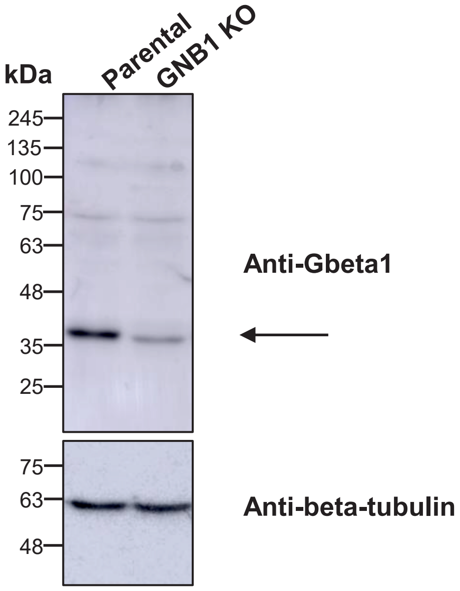

Supplemental Figure S2

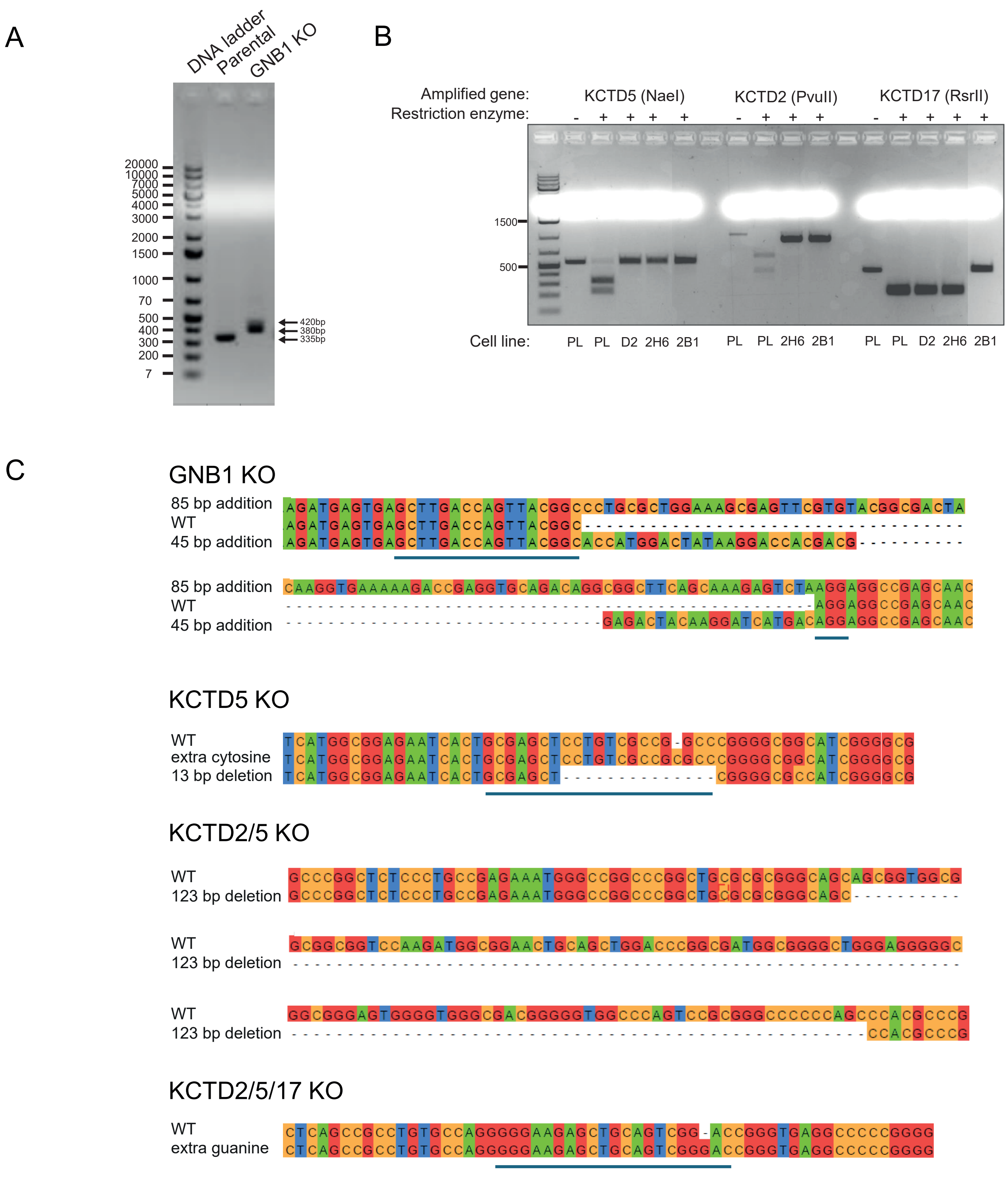

Supplemental Figure S3

A

B

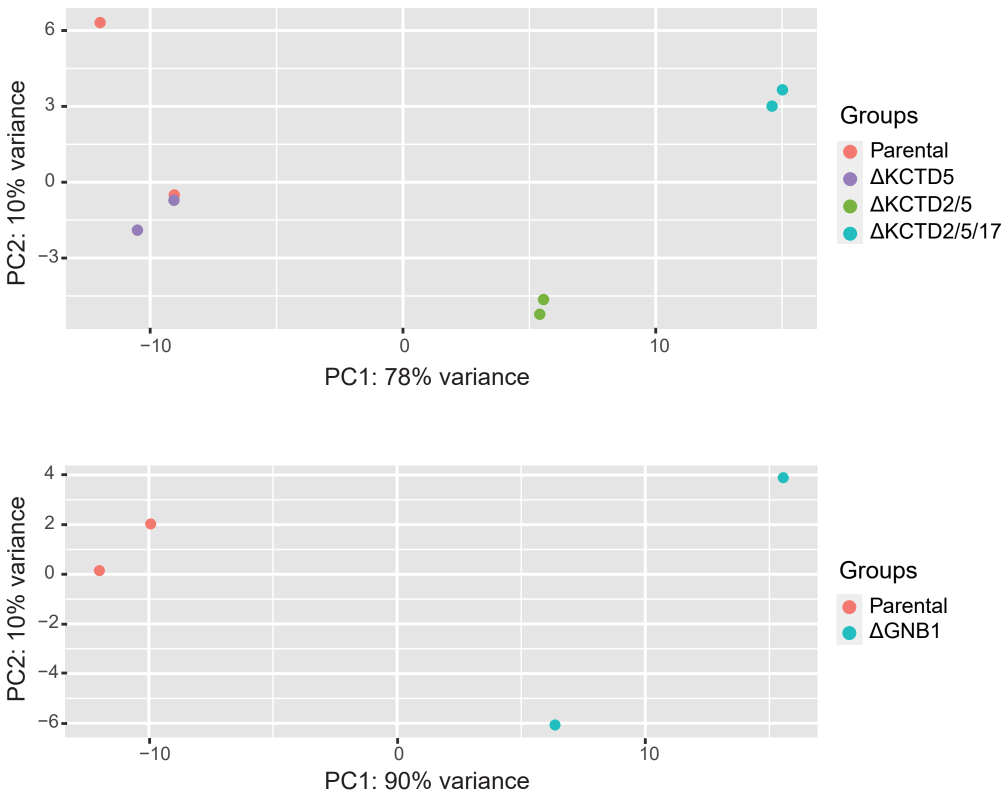

Supplemental Figure S4

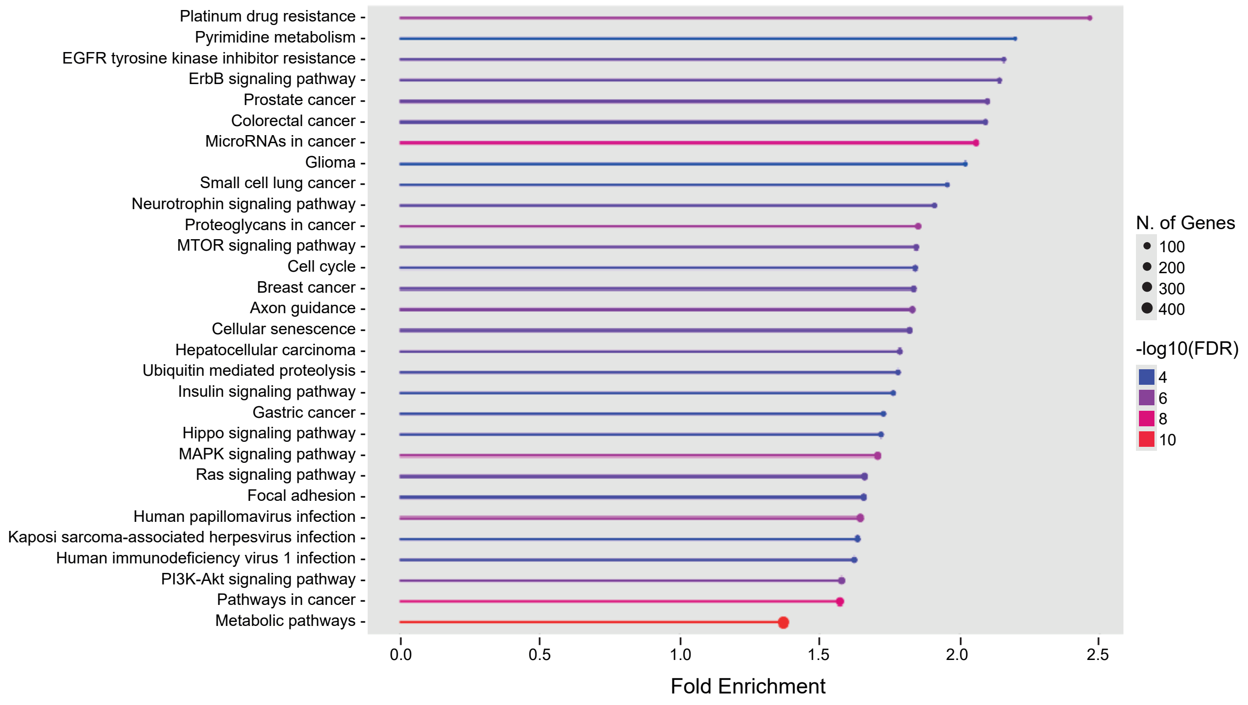

Supplement: Supplementary file 1 [file ijms-25-04993-s001.zip › ijms-2921079-SI.pdf]
